# Pre-expanded occipito-dorsal flap reconstruction for neck burns: A novel approach

**DOI:** 10.4103/2321-3868.130193

**Published:** 2014-04-06

**Authors:** Sameena Hassan, Peter Brooks

**Affiliations:** Department of Plastics, Burns and Reconstructive Surgery, Nottingham University Hospitals, National Health Service Trust, City Campus, Hucknall Road, Nottingham, England

**Keywords:** Burns, neck, tissue expansion, occipito-dorsal flap

## Abstract

Reconstruction of the neck following a burn injury poses a significant challenge to reconstructive burn surgeons. Here, we report a case of successful application of pre-expanded occipito-dorsal flaps in the reconstruction of postburn scars and contractures in the neck. The patient was a 10-year-old boy who sustained scars and contractures secondary to a burn injury 4 years ago. “Super-thin” flaps were obtained through pre-expansion in the occipito-dorsal area and then transferred to the recipient site. This approach resulted in an esthetic satisfaction and a significant functional improvement, thereby having significant clinical implications in the reconstruction of soft tissue damage secondary to burn injuries in the neck.

## Introduction

Post-burn deformities or scar contractures in the head and neck region impact the victim more significantly than those in the other parts of the body, because of both functional and esthetic reasons. Reconstruction of this region poses a significant clinical challenge to burn surgeons. Currently, a variety of techniques are available to burn reconstructive surgeons, among which transfer of expanded microvascular flaps has perhaps become the most favored option worldwide.[1] This technique often allows for reconstruction of large areas with good quality transposition or rotational flaps with a near-perfect match in skin color and texture, thus generating excellent aesthetic results. Nevertheless, expanded free flap transfer is associated with numerous complications include infection, extrusion, hematoma, flap ischemia and expander perforation.[2–4]

**Table 1:** 

Access this article online	
**Quick Response Code**:	**Website**: www.burnstrauma.com
	**DOI**: 10.4103/2321-3868.130193

Transfer of “super-thin” flaps expanded from the occipital, clavicular, or pectoral area has been previously described as an alternative approach to minimize the risk of complications associated with the conventional reconstruction techniques. [5] Here we report a case of successful reconstruction of postburn scars and contractures in the neck using pre-expanded occipital dorsal flaps.

## Case report

**Figure 1:**
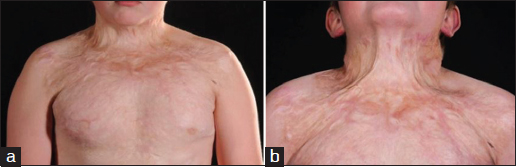
A photo of the patient before reconstruction, showing postburn scars and contractures. (a) Neck in neutral position. (b) Neck in extended position.

A 10-year-old boy presented to our unit with significant scars, keloids, and contractures in his anterior chest and neck that were formed secondary to burn injuries 4 years ago [Figure 1]. With a past history of asthma, he was on inhalers on an occasional basis. Having been experiencing disfigurement-related emotional and psychosocial abuse after burn injuries, he expressed a strong desire to have his original appearance restored. Accordingly, a two-stage reconstructive procedure was performed.

**Figure 2:**
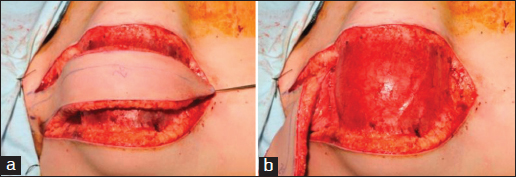
A photo of the elevating of the expanded flap, showing the occipital arteries and the envelope flap raised on a narrow isthmus with occipital arteries marked on it. (a) The expanded flap raised on a narrow isthmus with occipital arteries marked on it. (b) The envelope formed around the expander.

Given severe hypertrophic scars in his pectoral and supraclavicular areas resulting from the original burn injury, the occipito-dorsal area was the only available and appropriate donor site of local autologous skin for neck reconstruction. Accordingly, bilateral pre-expansion of occipito-dorsal flaps was conducted. Under general anaesthesia in the prone position, the shape of the flap was designed in a way to match the recipient site and the occipital arteries were identified and marked on the skin using a portable Doppler ultrasound machine [Figure 2]. Tissue expanders were positioned in a suprafascial plane inferior to the pedicle and deep to the center of the earlier defined occipito-dorsal flap, and the skin was closed directly. The expanders were inflated over a period of 3 months to a volume of 700 ml each. Prior to raising the flap, the occipital arteries were again dopplered and the flap was raised on a narrow isthmus, with precautions taken to ensure the flap remained above the fascial layer over the posterior trunk musculature. Once elevated, the flap was thinned to the layer in which the subdermal vascular network became visible through a layer of fat. Although designed as bilateral flaps, the two cavities converged as one. Upon rising, the expanders were removed. The donor site, measured 20 cm by 15 cm, was closed directly without drains. No significant donor site deformity was observed.

**Figure 3:**
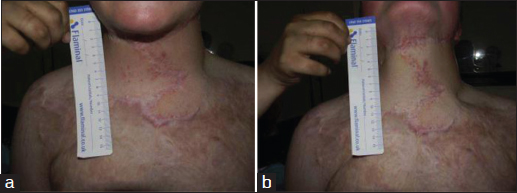
A photo of the patient after reconstruction, showing a satisfactory esthetic result. (a) Neck in neutral position. (b) Neck in extended position.

Two revision procedures were performed; the first one to address the dog ears in the posterior cervical region and the second one to remove excess adipose tissue by liposuction. As shown in Figure 3, these procedures achieved a satisfactory cosmetic result in terms of skin contouring, despite minimal scar hypertrophy. Moreover, these procedures resulted in a significant functional improvement; the patient was able to flex and extend the neck freely with no restrictions in lateral rotation.

## Discussion

Tissue expansion is an effective method for replacing scarred tissue secondary to burn insult. Being increasingly applied in reconstruction of the head and neck, this technique enables generation of flaps with a good cosmetic color match and reliable vascularity of an axial or random pattern.

The occipito-cervico-dorsal flap that is based on a descending branch of the occipital artery and arises close to the junction of rectus capitis and trapezius has been described previously.[5] In this flap, the occipital artery, transverse cervical artery, circumflex scapular artery, and dorsal intercostal perforator artery are all closely related, but have distinct anatomical territories which can be visualized with a hand-held Doppler device. Although distal partial necrosis may occur in larger flaps and thus restrict the size of the donor tissue that can be raised,[6] modification of the pre-expansion procedure performed in this case study significantly improved primary closure of the donor wound and the post transfer vascularity in the flap. Moreover, with the help of a portable Doppler, the extent of dissection required can be minimized.

In general, our unit prefers using expanded supraclavicular or pectoral skin whenever available, since it allows for vertical transfer rather than pedicle rotation. However, pectoral and supraclavicular skin was heavily scarred due to the original burn injury in the patient reported in this paper, and occipito-dorsal flap was the only source of local autologous skin for neck reconstruction. To avoid possible failure of the distal flap associated with aggressive thinning, we adopted a delayed liposculpture approach. Additionally, we utilized tissue expansion that might have conditioned the flap vasculature, thus increasing the chance of a successful outcome. It would be our interest to evaluate the possibility and benefits of using a central skin bridge to ensure the expander pockets remain separate in future.

In summary, this case report suggests that pre-expanded occipito-dorsal flap is worth considering when supraclavicular skin is not available for neck reconstruction. Although the skin on the back may be thick in some individuals and thus impose a limitation to the application of this flap, flap thinning using liposuction technique may be an effective solution. Nevertheless, patients should be counseled on the risks of tissue expansion and skin necrosis.
